# Improved Vision-Based Detection of Strawberry Diseases Using a Deep Neural Network

**DOI:** 10.3389/fpls.2020.559172

**Published:** 2021-01-11

**Authors:** Byoungjun Kim, You-Kyoung Han, Jong-Han Park, Joonwhoan Lee

**Affiliations:** ^1^Division of Computer Science and Engineering, Jeonbuk National University, Jeonju, South Korea; ^2^Horticultural and Herbal Crop Environment Division, National Institute of Horticultural and Herbal Science (RDA), Jeonju, South Korea

**Keywords:** strawberry diseases, cascade detector, deep neural network, detection, plant domain knowledge

## Abstract

Detecting plant diseases in the earliest stages, when remedial intervention is most effective, is critical if damage crop quality and farm productivity is to be contained. In this paper, we propose an improved vision-based method of detecting strawberry diseases using a deep neural network (DNN) capable of being incorporated into an automated robot system. In the proposed approach, a backbone feature extractor named PlantNet, pre-trained on the PlantCLEF plant dataset from the LifeCLEF 2017 challenge, is installed in a two-stage cascade disease detection model. PlantNet captures plant domain knowledge so well that it outperforms a pre-trained backbone using an ImageNet-type public dataset by at least 3.2% in mean Average Precision (mAP). The cascade detector also improves accuracy by up to 5.25% mAP. The results indicate that PlantNet is one way to overcome the lack-of-annotated-data problem by applying plant domain knowledge, and that the human-like cascade detection strategy effectively improves the accuracy of automated disease detection methods when applied to strawberry plants.

## Introduction

Effectively protecting plants from diseases is a critical means of improving productivity and enhancing crop quality (Fuentes et al., 2018). The traditional methods for the identification and diagnosis of plant diseases - visual analysis by professional farmer or inspection of a sample in a laboratory - generally require extensive professional knowledge and high costs. Moreover, neither method is particularly effective, with carrying a high probability of failure in successfully diagnosing specific diseases, leading to erroneous conclusions and treatments (Ferentinos, 2018).

Detecting plant diseases in their earliest stages can reduce the need to rely on potentially harmful remedial chemicals and lower labor costs. As many greenhouses are quite large, it is not always easy for even the most experienced farmers to identify plant diseases before they have spread. For this reason, an automated disease detection process will prove to be a valuable supplement to the labor and skill of farmers.

Researchers have already developed automatic identification and diagnosis methods capable of reaching fast, convenient, and accurate conclusions based on image analysis and machine learning techniques. Currently, there are two types of vision-based disease detection methods. First, are those methods that require humans to be kept in the detection loop. For example, the PlantVillage dataset (Hughes and Salathé, 2015) consists of images of detached leaves. When a detection system trained with a dataset like this is installed on handheld mobile devices equipped with a camera, a human is expected to take the initiative in identifying suspicious leaves and running the app. In the second type of system, crop monitoring robots capable of surveilling the whole of a crop at once are installed throughout a greenhouse. The images collected by these robots can be analyzed to identify a potential disease and automatically alert the appropriate supervisor. While such a surveillance system presents many logistical challenges in migrating from the drawing-board to the greenhouse, detection model is highly desirable for its superior early detection abilities and potential for cost savings.

The typical farmer approaches disease detection in two stages. First, he or she tries to identify suspicious areas on a plant that, based on his (or her) domain knowledge, may indicate disease. They then must determine whether it is a real disease or not. This paper proposes an improved deep learning–based strawberry disease detection method that relies on automatic visual object detection to imitate the two-steps of the human expert–detection process. First, our proposed system uses domain knowledge specific about plants for transfer learning. Second, it relies on a cascade detection scheme, by which it first glances over the objects in order to identify large suspicious regions, and then scrutinizes the suspicious area more closely to precisely identify the disease.

In general, plants vary in the shapes and colors of their flowers, leaves, stems, fruits, and roots. In light of this great diversity, it is inappropriate to analyze and extract plant features using a pre-trained model based on the ImageNet dataset, as this dataset does not reflect adequate domain knowledge. Typically, source and target domains are well connected with each other, which maximizes the effect of transfer learning. This explains why it is so inefficient to use pre-trained models with coarse-grained public datasets like ImageNet (Krizhevsky et al., 2012) for plant disease detection or classification.

Our proposed method uses networks named PlantNets, pre-trained with the PlantCLEF dataset from the LifeCLEF 2017 challenge (Joly et al., 2017), to construct a deep object detection model by transfer learning. Unlike prior disease detection or classification approaches (Kawasaki et al., 2015; Mohanty et al., 2016; Sladojevic et al., 2016; Brahimi et al., 2017; Cruz et al., 2017; Fuentes et al., 2017, 2018, 2019; Ramcharan et al., 2017, 2019; Barbedo, 2018; Ferentinos, 2018; Liu et al., 2018; Nie et al., 2019; Too et al., 2019), we assume a PlantNet backbone network pre-trained on the PlantCLEF dataset has the low-level domain knowledge of a farmer experienced at plant and disease identification.

The proposed method then uses a two-stage cascade detection model. In the first stage, the system surveilles a large visual field, seeking visual symptoms of diseases or other objects that may possibly cause a false positive. In the second stage, the system closely checks only the disease-suspected area to improve diagnosis and precision. In this way, the system reduces false positives caused by adverse environmental factors, like glare or other objects.

In our experimental a single-stage detector with a PlantNet provided an 86.4% mAP in the complex task of strawberry disease detection, which represents a 3.27% improvement over a model pre-trained on the ImageNet dataset. In addition, the cascade model produced approximately 91.65% mAP, which represents a 5.25% gain over a non-cascade model.

The remainder of this paper is organized as follows. In the next section, we provide a detailed review of previous works related to our method. Further details about the cascade strategy for disease detection with a PlantNet are provided in Section 3. In Section 4 we outline the proposed approach to strawberry disease detection. Our experimental results are presented in Section 5, along with a discussion of the performance of our method. Finally, in Section 6 we summarize our work, and suggest what new lines of inquiry have been opened by our research.

## Related Works

A conventional computer vision approach consists of feature exploration by image analysis and identifier construction by machine learning. Many visual feature classifiers have been developed for application in many problem domains (Zhao et al., 2019). In general, for traditional computer vision, there have been many expert-designed features descriptors developed, including the Scale Invariant Feature Transform (SIFT) (Lowe, 2004), the Haar (Viola and Jones, 2001), and the Histogram of Oriented Gradients (HOG) (Dalal and Triggs, 2005). Theses have been exploited with machine learning algorithms, such as the Support Vector Machine (SVM), to construct detectors or classifiers.

The deep learning approach differs from the conventional computer vision–based disease recognition in several way. First, the features are not selected by an expert, but are automatically derived by a network relying on a large amount of training data. In designing this approach, therefore, the goal is not to identify the proper features, but rather to finding an appropriate network and preparing suitable training data. Another difference is that the detector or the classifier can be obtained by simultaneously training it with features in the same deep neural network. Therefore, the appropriate network structure becomes more important with an efficient training algorithm.

There are two different vision-based approaches to plant disease recognition by machines: classification and detection. The classification approach relies on a human to visually identify symptoms of a plant disease, and then capture an image and ask a computer to provide a diagnosis. This approach starts with an image of part or whole of a plant canopy, diseased or healthy, and then follows this with an attempt determine whether the image includes symptoms of a specific disease.

The detection approach, in contrast, first tries to identify the location of symptoms, usually within bounding boxes, and classify them as specific diseases. This process is localized to, suspicious areas to reduce the necessity of human intervention. In this system, an image in which symptoms are detected results in an output of bounding boxes with the corresponding names of diseases. Input images for the detection approach are generally less restricted than those of the classification approach, as they can be captured from any direction or distance to reveal the symptoms. The detection approach can also be applied to a sequence of video frames, while the classification approach typically relies on only a single image. Being the more flexible and comprehensive of the two options, it comes as no surprise that a detection system was preferred in designing our automated system.

### Classification Approaches for Disease Identification

There have been a number of prior Deep Neural Network (DNN)-based classification approaches to plant diseases and disorder identification. Such DNNs usually consists of a multilayer Convolutional Neural Network (CNN)-based feature representation block which is followed by a classification block. The PlantVillage dataset has been widely used to train classifiers because of its excellent images of plant disease.

Table 1 summarizes recent research in which Deep Learning (DL)-based classification approaches have been applied. Note that a great deal of the research used the ImageNet pre-trained backbone models as a feature extractor, and the PlantVillage dataset to fine-tune the specific feature extractor and classifier to plants and diseases. In addition, (Park et al., 2018) proposed a DL-based classification approach for the five types of apple leaf conditions in hyperspectral images for diagnosis of Marssonina blotch, in which they selected several bands by minimum redundancy maximum relevance (mRMR). Also, (You and Lee, 2020) developed a lightweight MobileNet-based DNN for classifying citrus diseases and pests.

**TABLE 1:** L-based classification approaches for identification of plant diseases.

Authors	Network Models	Dataset for pre-training	Plants	Dataset for fine-tuning	Disease classes
Sladojevic et al., 2016	CaffeNet	ImageNet	15 spices	Collected from Internet	13
Cruz et al., 2017	Modified LeNet	PlantVillage	olives	PlantVillage	3
Mohanty et al., 2016	AlexNet, GoogLeNet	ImageNet	14 spices	PlantVillage	38
Barbedo, 2018	GoogLeNet	ImageNet	12 spices		12
Too et al., 2019	VGG16, InceptionV4, ResNet50, -101, -152, DenseNet121	ImageNet	14 spices	PlantVillage	38
Brahimi et al., 2017	AlexNet, GoogLeNet	ImageNet	9 diseases	PlantVillage	9
Ramcharan et al., 2017	InceptionV3	ImageNet	cassava	Collected from fields	5
Kawasaki et al., 2015	Modified LeNet		cucumber	Collected from laboratory	3
Ferentinos, 2018	AlexNet, GoogLeNet, OverFeat, VGG16, AlexNetOWTBn		25 species	PlantVillage	58
Liu et al., 2018	AlexNet	ImageNet	apple	Collected from fields	4

### Detection Approaches for Disease Identification

In general, DL-based visual object detections are divided into single-step and multi-step approaches. OverFeat (Sermanet et al., 2013) was one of the first single-stage object detections based on deep learning networks. There are two primary means of applying the single-step approach; Single Shot Multi-Box Detector (SSD) (Liu et al., 2016) and You Only Look Once (YOLO) (Redmon et al., 2016). The single-step approach reduces the computational complexity of the network to improve detection speed, which is its primary advantage over the multi-step approach. Plant disease detections by DL-based computers that use the single-step approach have been developed for various spices, such as cassava (Ramcharan et al., 2017, 2019) and tomatoes (Fuentes et al., 2017, 2018, 2019).

The multi-step approach is based on a two-step process. The first step generates a set of candidate region proposals (Uijlings et al., 2013), and the second step classifies the proposals into either foreground (enclosed by bounding boxes) or background. This approach originated from the Region-based Convolutional Neural Network (R-CNN) (Girshick et al., 2014) and successive improvements in detection speed and accuracy led to the Fast R-CNN (Girshick, 2015), the Faster R-CNN (Ren et al., 2015), the Region-based Fully Convolutional Network (R-FCN) (Dai et al., 2016), and the Feature Pyramid Network (FPN) (Lin et al., 2017). Several plant disease detection models have used the multi-stage approach to identify, for example, verticillium wilt in strawberries (Nie et al., 2019), and diverse diseases in tomatoes (Fuentes et al., 2017, 2018, 2019). Table 2 summarizes plant disease identification based on visual object detection.

**TABLE 2:** Summary of visual object detection models for plant disease monitoring.

Authors	Models	Dataset for pre-training	Plants	Dataset for fine-tuning	No. of classes
Nie et al., 2019	Faster R-CNN + Attention	ImageNet	strawberry	Collected from fields	4
Ramcharan et al., 2019	SSD	MSCOCO	cassava	Collected from fields	3
Fuentes et al., 2017	Faster R-CNN,R-FCN, SSD	ImageNet	tomato	Collected from fields	9
Fuentes et al., 2018	Faster R-CNN + FilterBank	ImageNet	tomato	Collected from fields	10
Fuentes et al., 2019	FPN + LSTM	ImageNet	tomato	Collected from fields	10

As gathering and labeling data for disease identification is expensive, all of the prior research used pre-trained networks and applied transfer learning. Gathering even annotated data for object detection is much harder than it is for classification, as bounding boxes must be provided for ground truth. In general, all DNN-based object detection methods adopted the backbone networks pre-trained on the ImageNet dataset of the ImageNet Large Scale Visual Recognition Challenge (ILSVRC) or the Microsoft Common Objects in Context (MS COCO) dataset (Lin et al., 2014), which is appropriate for coarse-grained identification tasks. Because there is no public dataset like PlantVillage for classification, every detection model developed has used its own dataset collected from the field to fine-tune the network.

## The Proposed Approach

In light of the many advantages of the object detection model for automating early monitoring for plant diseases, our DL-based approach adopts this approach. In our two-stage cascade structure, the initial diagnosis stage identifies suspected diseased area and the second stage is used for fine detection. Each stage is constructed with one of the PlantNets as a backbone for the feature extractor, which is pre-trained using the PlantCLEF dataset from the LifeCLEF 2017 challenge.

The scope of this work does not include abiotic stresses. We assume that strawberry growing in relatively small sized greenhouses in South Korea, where the abiotic stresses are relatively rare and the image capturing is less hard to be incorporated with a robot system.

### Plant Domain Knowledge Network (PlantNet)

Detecting disease on the basis of plant images is not a coarse-grained task like ILSVRC, which includes objects such as elephants, cars, apples, etc., and which all look different from each other. Many plant diseases and disorders look very similar, so even an expert might struggle to correctly diagnose and remediate the symptoms. Accordingly, the backbone networks pre-trained with an ImageNet dataset may not be sufficient to be applied to the tack of detecting disease from plant images.

Successful transfer learning requires that, the visual features in the target domain should be properly transformed from those of the source domain by fine-tuning, even with a small training dataset. Labeled data for plant disease detection is expensive to acquire, as the name and location of a disease must be annotated with the corresponding bounding box by a domain expert. Accordingly, it was inevitable that we would have to overcome a lack-of-training-data problem in our efforts to achieve accurate detection.

Because no large image dataset for plant diseases exists, we relied on the PlantCLEF dataset from the LifeCLEF 2017 challenge, which was designed to study biodiversity and agrobiodiversity (Joly et al., 2017). We believe PlantCLEF is superior to ImageNet for extracting the proper features of plant diseases, as these images are particularly well-suited successfully fine-tune for plant disease detection with a small amount of domain-specific, annotated training data.

**FIGURE 1:**
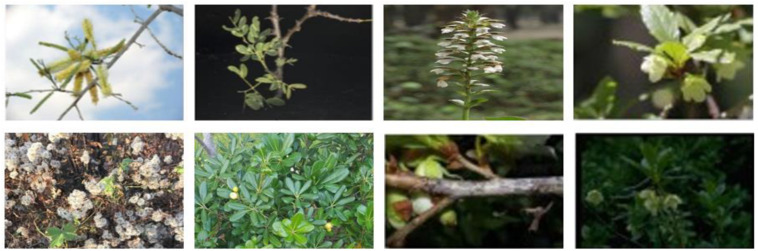
Examples of noiseless samples from the PlantCLEF dataset in LifeCLEF 2017 challenge (Joly et al., 2017).

The PlantCREF dataset includes images of weeds, trees, ferns, etc., and their various parts, including the flowers, fruits, and leaves. The dataset includes 256,278 credible and noiseless plant images, and approximately 1.45M images with noise gathered off the internet. Figure 1 shows several sample images taken from the noiseless dataset. We filtered out thousands of noisy images, and retrained approximately 1.25M images.

We tested three recent backbone networks, including ResNet50, ResNet152, and the InceptionResNet v2 models, to do the classification task of the LifeCLEF 2017 challenge. We ultimately choose ResNet152, which gave the best performance with respect to Top-5 classification accuracy.

### Cascade Strawberry Diseases Detection Model

**FIGURE 2:**
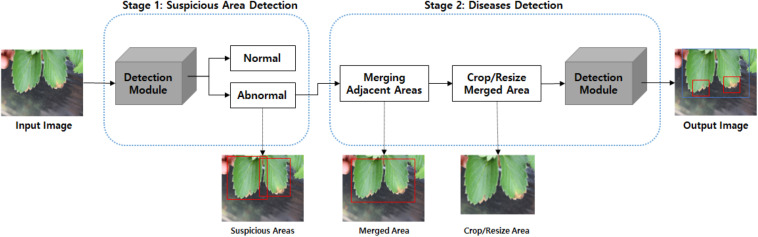
Proposed strawberry disease detection model.

**FIGURE 3:**
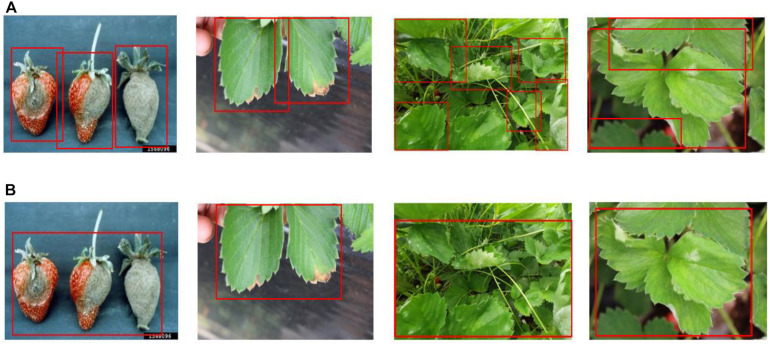
Detection and merged results of the first stage: **(A)** suspicious areas of disease in the first stage, and **(B)** merged areas for input to the second stage. [Forestry Images (2020); photographer: Sikora].

Our strawberry disease detection model adopts a two-step cascade object detection technique. Figure 2 shows the proposed detection model. The first stage detects suspicious areas of diseases with a low threshold-of-confidence score for the locations of the bounding boxes. In general, the role of the first stage in cascade detection is to increase the recall rate by using a low detection threshold. Improving precision and reducing false detections is the goal of the second stage. In the first stage, we defined three categories; “normal,” “abnormal (fruit, leaf),” and “background object” (to include items such as vinyl or other items that are not part of the plant). Figure 3 shows the detection and merged results of the first stage. Adjacent suspicious areas of disease (a short distance apart) were then merged as rectangles to prepare a single large area. In this merging process the detected background objects were excluded so that they would not be examined in the second stage. This process removed areas where diseases would not manifest in order to reduce false detections and reduce speed in reaching the second stage.

The merged area of the rectangle was cropped and resized to feed into the second stage module, where suspicious areas were again scrutinized to more precisely detect seven categories of six diseases, including “angular leafspot,” “anthracnose fruit rot,” “gray mold,” “leaf blight,” “leaf spot,” and “powdery mildew fruit/leaf.” The final results of the analysis of disease class and locations are displayed in the input image. The second stage review provides high accuracy and low false detection rates by carefully observing only the confined areas. Note that the structure of the detection modules are identical even though the object classes to be detected are different from each other.

### Strawberry Disease Detection Modules

**FIGURE 4:**
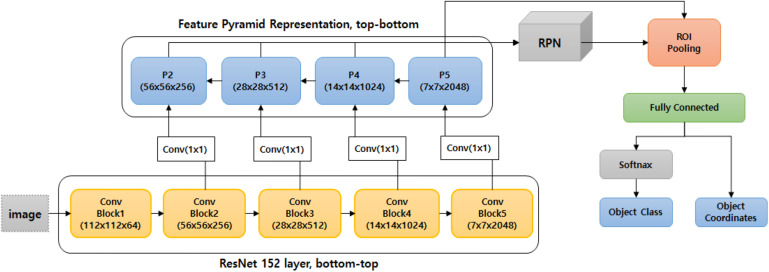
ResNet152 backbone and the FPN-structured disease detection module.

Because the ResNet152 backbone network was the best performer at the LifeCREF 2017 classification task, it was chosen as the initial backbone feature extractor for both the first and second detection modules. Once the features are extracted, they are captured in an FPN structure. Based on this multi-scale feature representation, the regions to be classified are proposed and precisely located. Figure 4 shows the ResNet152 backbone and the FPN-structured disease detection module.

## Experiments

In this section, we present the experimental results for the PlantNets, and our collected strawberry dataset.

### LifeCLEF 2017 Task and PlantNets

**FIGURE 5:**
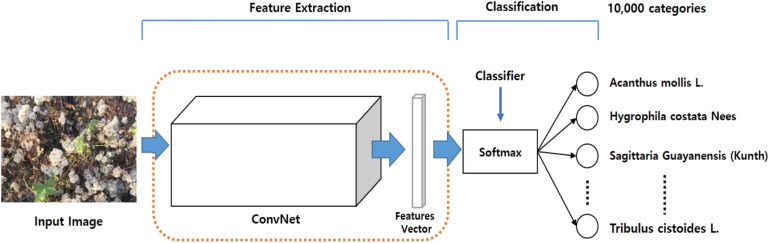
LifeCLEF 2017 task for a round dotted red-rectangle PlantNet.

To demonstrate the necessity for transfer learning, and to identify the backbone network best suited to capturing visual features from plant images, we followed the LifeCLEF 2017 task, which constructs an image classifier for 10,000 categories of different plants, as shown in Figure 5.

In our experiment, we first comprise the backbone networks with the parameters trained on the ImageNet dataset and adjusted the parameters associated with the Softmax classifier for the 10,000 categories. Note that this experiment relied on only the learned feature extractor that was trained on the ImageNet dataset, and only attempted to train the classifier to perform the task. This setting resulted in poor performance, as expected, by the ResNet50, ResNet152, and InceptionResNetV2, as listed in Table 3.

**TABLE 3:** Summary of results from the LifeCLEF 2017 Task.

Backbone	Accuracy	Features obtained from ImageNet dataset	Fine-tuned by PlantCLEF 2017 dataset	
			
			250K noise-free data	1.50M + noisy data
ResNet50	Top-1	20.80%	51.70%	69.60%
	Top-5	26.30%	79.50%	85%
ResNet152	Top-1	27.10%	64.80%	76.30%
	Top-5	32.80%	84.00%	91.00%
InceptionResNetV2	Top-1	24.50%	67.50%	75.50%
	Top-5	38.00%	83.50%	90.00%

The backbone was then pre-trained with the ImageNet dataset and the Softmax classifier was fine-tuned with the LifeCLEF 2017 dataset. Performance was greatly improved by fine-tuning both the backbone feature extractor and the classifier network, suggesting that fine-tuning is necessary prerequisite to improving performance with domain-specific data. Performance improved as the amount of data increased, even when noisy data was included. Among the Top-5 categories, ResNet152 was the best among the three, providing 91.0% classification accuracy, as shown in Table 3. Based on these results, we chose ResNet152 as the backbone of the strawberry disease detector. The dotted red rounded rectangle in Figure 5 represents the PlantNet.

### Strawberry Dataset

**FIGURE 6:**
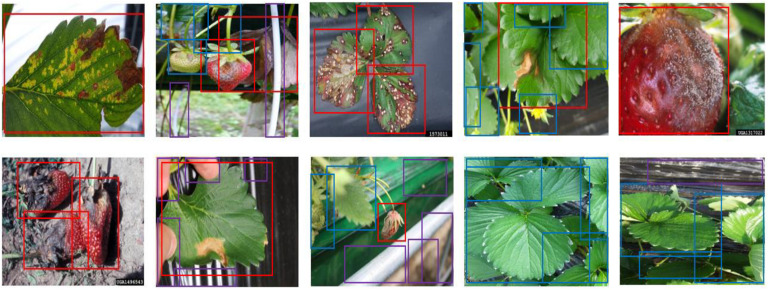
The annotated sample images from the collected dataset. Ground truth is annotated by bounding boxes with the following class categories: diseases (red box), normal plants (blue box), and background object (purple box).

We collected 4,560 images from different greenhouses using camera-equipped mobile phones, and combine them with 175 Forestry Images^1^ for a total 4,735 images as in Table 4. We assumed that the images were collected from diverse environmental conditions for strawberries, as they were taken different farms. The dataset included images of the beginning, middle, and final stages of various diseases, as well as unblemished strawberries. Such diversity in the dataset is important to successfully train a generalized disease detector. Figure 6 shows sample images from the dataset.

**TABLE 4:** Number of images for strawberry disease detection.

Categories of positive and negative examples	Original number of images	Images for training and validation	Augmented number of images for training and validation	Numbers of test images for evaluation
Angular leafspot	319	223	2676	96
Anthracnose fruit rot	231	161	1932	70
Gray mold	235	164	1968	71
Leaf blight	294	209	2508	85
Leaf spot	593	415	4980	178
Powdery mildew fruit	137	97	1164	40
Powdery mildew leaf	718	502	6024	216
Normal	2000	1400	16800	600
Background	198	138	1656	60
Total	4725	3309	39708	1416
				

### Training the Strawberry Disease Detector

The dataset was divided into training, validation, and test datasets. About 60% of the images in the dataset were taken for training, and 10% were used for validation. To reduce the chance of overfitting, we augmented the training and validation dataset with geometric transformations, including resized, cropped, rotated, horizontal/vertical flipped, and PCA color-changed images. The test dataset used evaluation was not augmented. Table 4 describes the training, validation, and testing datasets in greater detail. We separated the background, which included objects like pillars that supported the greenhouse and black vinyl to protect against weeds, from the foreground images of the strawberry plant. The normal category includes images of healthy strawberries, which corresponds to the negative examples as well as background in the first stage of cascade detection. Seven different categories of strawberry diseases were considered (powdery mildew has two different categories, depending on where it manifests on the plant). As shown in Figure 6, ground truth is annotated by bounding boxes with the following class categories: diseases (red box), normal plants (blue box), and background object (purple box). From the images listed in Table 4, we annotated 21,252 bounding boxes for abnormal classes (positive examples of diseases and background objects) and 16,800 for normal classes (negative examples, i.e., normal strawberries).

Training was performed separately for each detection module. Because this includes merging the bounding boxes and the crop/resizing process that prepares the input for the second stage of the detection module, end-to-end learning was not possible. The proposed model was trained and evaluated on an Intel Xeon CPU E5-2650, 64GB RAM, and a single NVidia Geforce TiTanXP GPU. The model parameters were selected as follows: the maximum number of iterations was 250,000, the initial learning rate of 0.001 with the decaying rate of 0.1 for 80,000/150,000 iterations, weight decay was 0.00004, momentum rate of 0.9. During the training, OHEM (Online Hard Example Mining) was adopted to effectively relieve the class imbalance problem and decrease the false positive error.

### Evaluation Method

The performance of the proposed model was evaluated based on average precision (AP) introduced by the Pascal VOC Challenge (Everingham et al., 2010). AP is the area under the precision and recall curve for the detection task, and has a constant recall level of 0 to 1. The equation for AP is as follows:

(1) $$\text{AP=}\frac{1}{11}\sum_{\text{r}\in \text{\{0,0}.1,0.2,...\text{,1\}}}{\text{P}}_{\text{interp}(r)}$$

(2) $$P_{i\,n\,t\,e\,r\,p}\,\left(r\right)=m\,a\,x_{\tilde{r}:\tilde{r}\geq r}\,P\,\left(\tilde{r}\right)$$

where *P*_*interp*_(*r*) and $P\,\left(\tilde{r}\right)$ represent the maximum precision for any recall values greater than r, and the measured precision of recall, $\tilde{r}$, respectively. The intersection over union (IoU) defined in Equation 3, is a used method for evaluating the detector accuracy:

(3) $$\text{IoU}\,\left(A,B\right)=\left|\frac{\text{A}\,\cap \text{B}}{\text{A}\,\cup \text{B}}\right|$$

where *A B*represent the ground truth box collected in the annotation and *B* represents the predicted result of the network. If the estimated IoU was higher than a given threshold, the predicted results were considered as positive samples (TP + FP) otherwise they were considered negatives (FN + TN). IoU is a parameter where the bounding box detected is used to identify True Positive(TP), True Negative(TN), False Positive(FP), and False Negative(FN).

## Experiment Results and Discussion

### ImageNet and PlantNet Pre-trained Backbone in a Single Stage Detector

To determine the effect of PlantNet on performance, we compared two different types of backbone feature extractors in a single-stage detector; one was a ResNet152 pre-trained only on the ImageNet dataset and the other was a ResNet152 pre-trained on a PlantNet obtained from the LifeCLEF 2017 task. For both experiments, the single-stage detectors were adjusted and fine-tuned with the strawberry training dataset. With the same detection threshold of 0.5 for the FPN, pre-training with ImageNet resulted in 83.13% mAP, while pre-training from PlantNet resulted in 86.4% mAP. This result clearly shows how direct use of a PlantNet pre-trained backbone is superior to an ImageNet. PlantNet is capable of capturing domain knowledge prior to being fine-tuned with a small amount of domain-specific data on strawberry diseases. Table 5 summarizes the results of the experiment.

**TABLE 5:** Comparison of results from ImageNet- and PlantNet-pre-trained backbones in a single-stage detector.

Class	Average precision of the ImageNet pre-trained backbone	Average precision of PlantNet
Angular leafspot	74.72%	81.69%
Anthracnose fruit rot	87.38%	90.23%
Gray mold	89.68%	90.00%
Leaf blight	87.64%	89.81%
Leaf spot	71.66%	80.48%
Powdery mildew fruit	83.94%	84.01%
Powdery mildew leaf	86.91%	88.59%
mAP	83.13%	86.40%

### ImageNet and PlantNet Pre-trained Backbones in a Cascade Detector

In the experiment, we constructed a cascade detector with two different types of backbone feature extractor; one pre-trained with the ImageNet dataset, and the other pre-trained with PlantNet. The number of bounding boxes detected by the object detector decreased as the IoU threshold increased. However, the impact on the cascade structure should also be considered in terms of the recall rate, which is equal to TP/(TP + FN). Table 6 shows true positive, false negative and recall for the IoU detection threshold in the first stage.

**TABLE 6:** Comparison of results from the IoU detection threshold in the first-stage detector.

IoU threshold	True Positive	False Negative	Recall
0.1	39708	0	1
0.2	39578	130	0.9941976
0.3	39492	216	0.989226633
0.4	39333	375	0.983649467
0.5	38160	1548	0.961471133

IoU detection threshold for the second stage was 0.5 in order to increase precision. The overall mAPs for the ImageNet was 88.05% and 91.65% for the PlantNet pre-trained backbones, which are at least 4.92% and 5.25% higher than those from the single-stage detector.

**FIGURE 7:**
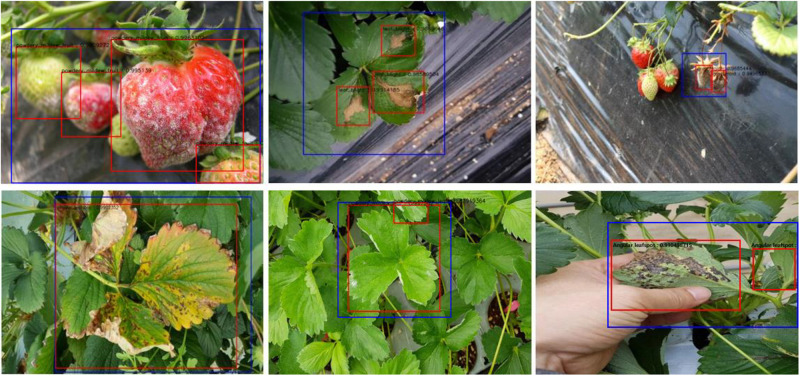
Samples of the final detected results.

**FIGURE 8:**
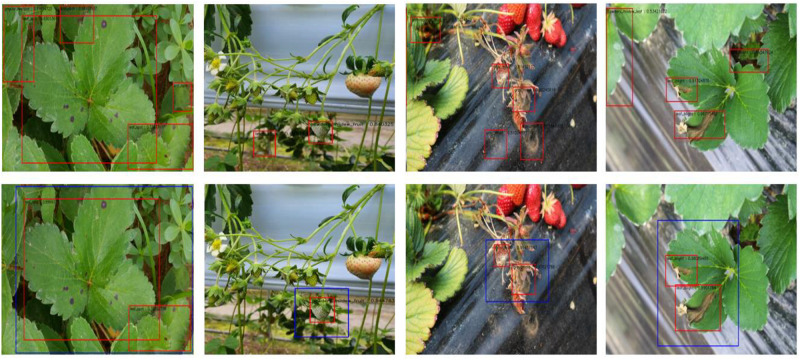
Comparison of the detection results between the single stage **(upper row)** and two-stage cascade detector **(lower row)**.

The average time until disease detection was 0.241 s for the single stage cascade detector and 0.662 s for the two-stage cascade detector, using the computation resources described in Section “Training the Strawberry Disease Detector.” This shows that the cascade structure improves accuracy, though it comes at the cost of increased detection time. In order to speed up the detection process, pipeline processing could be adopted with our cascade approach. Table 7 summarizes our results, and Figure 7 shows samples of our detection tests, in which blue rectangles represent the merged suspicious areas of disease, and the red boxes are the diseases detected in the second stage. Figure 8 compares the results of the single stage and the cascade detectors. The false positive errors in the upper row of the single stage detector are effectively reduced in the lower row of the two-stage cascaded detector.

**TABLE 7:** Comparison of the results from ImageNet and PlantNet pre-trained backbones in the cascade detector.

Class	Average precision of ImageNet pre-trained backbone	Average precision of PlantNet
Angular leafspot	88.41%	94.55%
Anthracnose fruit rot	90.23%	96.01%
Gray mold	90.17%	94.52%
Leaf blight	89.81%	91.82%
Leaf spot	85.16%	87.83%
Powdery mildew fruit	84.01%	86.36%
Powdery mildew leaf	88.59%	90.48%
mAP	88.05%	91.65%

It is difficult to compare the performance of our proposed method using relative terms, as we have attempted to detect different set of diseases with different data than other researchers. For example, (Nie et al., 2019) have achieved 99.95% accuracy in identifying four diseases, while others (Kusumandari et al., 2019; Shin et al., 2020) have sought to identify only a single disease.

## Conclusion

Protecting plants from diseases is critical to maximizing farm productivity and achieving higher crop quality. The earlier plant diseases can be detected, the more effective and targeted an intervention can be. We reviewed recent research into vision-based plant disease identification, especially research that involved use of DL. We then categorized these interventions into two types based on the degree of human intervention: the ‘classification’ and ‘detection’ approaches.

After determining that the detection approach was superior, we proposed an improved method of vision-based detection of strawberry diseases using a DNN that is capable of being incorporated into an automated robot system. In our approach, the backbone feature extractor PlantNet, which was pre-trained on plant data like the PlantCLEF dataset for the LifeCLEF 2017 challenge, was installed with a two-stage cascade disease detection model. PlantNet captured plant domain information quite well, and it demonstrated performance superior to that of the backbone pre-trained on an ImageNet-type public dataset by at least 3.2% mAP. The cascade detector also improved accuracy by up to 5.25% mAP. The results indicate that PlantNet is one way to overcome a lack of annotated data by applying plant domain knowledge, and that the human-like cascade detection strategy is effective at improving accuracy.

Diseases and abiotic stresses in the strawberry plant occur everywhere in greenhouses, which is related to environmental data such as low or high temperature, deficient or excessive water, high salinity, heavy metals, and ultraviolet radiation. These factors are hostile to plant growth and development, leading to crop yield penalty (He et al., 2018). For this reason, pursuing even more accurate, quick, and practical strawberry plant disease and abiotic stress lesion detection techniques is necessary. It can also be helpful to improve the visual analysis results by integrating other environmental data, such as humidity, temperature, and nutrition. In the future development of the proposed method, we will continuously collect environmental and strawberry plant image data for the disease and abiotic stress and can improve the detection accuracy by fusion of visual detection results and environmental knowledge information.

## Data Availability Statement

The original contributions presented in the study are included in the article/supplementary material, further inquiries can be directed to the corresponding author/s.

## Author Contributions

BK and JL designed the study, performed the experiments, data analysis, and wrote the manuscript. JL advised on the design of the model and analyzed to find the best method for Improve detection accuracy of diseases of strawberry plants. Y-KH and J-HP collected the data from the greenhouse and contributed the information for the data annotation.

## Conflict of Interest

The authors declare that the research was conducted in the absence of any commercial or financial relationships that could be construed as a potential conflict of interest.

## Footnotes

1 Forestry Images (2020), https://www.forestryimages.org/index.cfm (accessed November 7, 2020). Photographers: Brenda Kennedy, University of Kentucky, Bugwood.org; Bruce Watt, University of Maine, Bugwood.org; Clemson University – USDA Cooperative Extension Slide Series, Bugwood.org; Don Ferrin, Louisiana State University Agricultural Center, Bugwood.org; Edward Sikora, Auburn University, Bugwood.org; Florida Division of Plant Industry, Florida Department of Agriculture and Consumer Services, Bugwood.org; Gerald Holmes, Strawberry Center, Bugwood.org; Garrett Ridge, NCSU, Bugwood.org; Jonas Janner Hamann, Universidade Federal de Santa Maria (UFSM), Bugwood.org; Ko Ko Maung, Bugwood.org; Mary Ann Hansen, Virginia Polytechnic Institute and State University, Bugwood.org; Natalie Hummel, Louisiana State University AgCenter, Bugwood.org; Paul Bachi, University of Kentucky Research and Education Center, Bugwood.org; Rebecca A. Melanson, Mississippi State University Extension, Bugwood.org; Scott Bauer, USDA Agricultural Research Service, Bugwood.org; U. Mazzucchi, Università di Bologna, Bugwood.org.
